# Genome-Wide Analysis of the Binding of the Hox Protein Ultrabithorax and the Hox Cofactor Homothorax in *Drosophila*

**DOI:** 10.1371/journal.pone.0014778

**Published:** 2011-04-05

**Authors:** Siew Woh Choo, Robert White, Steven Russell

**Affiliations:** 1 Department of Genetics, University of Cambridge, Cambridge, United Kingdom; 2 Department of Physiology, Development and Neuroscience, University of Cambridge, Cambridge, United Kingdom; 3 Cambridge Systems Biology Centre, University of Cambridge, Cambridge, United Kingdom; Georgia Institute of Technology, United States of America

## Abstract

Hox genes encode a family of transcription factors that are key developmental regulators with a highly conserved role in specifying segmental diversity along the metazoan body axis. Although they have been shown to regulate a wide variety of downstream processes, direct transcriptional targets have been difficult to identify and this has been a major obstacle to our understanding of Hox gene function. We report the identification of genome-wide binding sites for the Hox protein Ultrabithorax (Ubx) using a YFP-tagged *Drosophila* protein-trap line together with chromatin immunoprecipitation and microarray analysis. We identify 1,147 genes bound by Ubx at high confidence in chromatin from the haltere imaginal disc, a prominent site of *Ubx* function where it specifies haltere versus wing development. The functional relevance of these genes is supported by their overlap with genes differentially expressed between wing and haltere imaginal discs. The Ubx-bound gene set is highly enriched in genes involved in developmental processes and contains both high-level regulators as well as genes involved in more basic cellular functions. Several signalling pathways are highly enriched in the Ubx target gene set and our analysis supports the view that Hox genes regulate many levels of developmental pathways and have targets distributed throughout the gene network. We also performed genome-wide analysis of the binding sites for the Hox cofactor Homothorax (Hth), revealing a striking similarity with the Ubx binding profile. We suggest that these binding profiles may be strongly influenced by chromatin accessibility and provide evidence of a link between Ubx/Hth binding and chromatin state at genes regulated by Polycomb silencing. Overall, we define a set of direct Ubx targets in the haltere imaginal disc and suggest that chromatin accessibility has important implications for Hox target selection and for transcription factor binding in general.

## Introduction

Hox genes play a key role in development as they are responsible for specifying the differences between segments along the body axis [1]; reviewed in [2]. Different Hox genes are expressed in overlapping patterns along the antero-posterior axis forming a Hox code that specifies particular target gene activities in each segment and hence generates specific segmental morphologies. The Hox system is highly conserved and appears to function in a very similar way across a wide range of metazoans to generate segmental diversity; for example, in specifying which segments carry legs in insects and which vertebrae carry ribs in vertebrates.

Although Hox genes have been studied for many years and their developmental roles are well characterised we still do not know, in any species, the sets of target genes they regulate [3], [4] or understand the molecular basis of their target specificity [5]. In Drosophila, some target genes have been identified; either through candidate approaches (e.g. [6]–[8]) or more systematic methods (e.g. [9]–[12]; reviewed in [4]) and for a small number of genes there is good evidence that they are direct targets (e.g. [6], [13]). It is important to systematically and comprehensively identify direct Hox targets for several reasons. First, analysis of in vivo binding is necessary to understand Hox target specificity; the Hox genes encode a set of closely related DNA-binding transcription factors that exhibit clear functional specificity in vivo but show little binding selectivity in vitro (reviewed in [5]). DNA binding specificity can be increased by interactions with cofactors, such as the homeodomain proteins Extradenticle (Exd; [14]–[16]) and Homothorax (Hth; [17]) but the in vivo roles of these cofactors have been controversial. At several target genes there is good evidence that cofactors contribute to binding specificity [18], at others the cofactors appear to modify Hox protein function [19], [20] and for some targets cofactors may not be required [21]. Second, to understand the interactions between Hox proteins and other regulatory inputs that enable, for example, Hox genes to regulate target genes appropriately in different tissues [22]–[24]. Third, to understand the gene networks that connect the Hox genes to the developmental processes that build particular segmental morphologies [11], [25]–[27].

Here we use Chromatin immunoprecipitation coupled with microarray analysis (ChIP-array) to identify direct targets of the Drosophila Hox protein Ultrabithorax (Ubx) and the Hox cofactor Homothorax (Hth). We have generated a high confidence set of Hox target genes which points to a wide range of processes under direct Hox control. In addition, our analysis of Ubx and Hth binding suggests a strong influence of chromatin accessibility in target selection.

## Results

### Generation of genomic binding profiles of Ubx and Hth

We used ChIP-array to investigate the genome-wide binding of Ubx and Hth. For this we have taken a tagged protein approach based on our previous experience using GFP-fusion proteins in ChIP studies [28], [29]. We identified protein trap lines from the Cambridge protein trap project, FlyProt [30], that contain YFP insertions into the endogenous Ubx and Hth transcription units. The FlyProt project generated a single line containing a YPF protein trap in the Ubx locus and 6 lines with insertions in hth. We screened these lines for suitability for use in ChIP array by examining expression and phenotype. The Ubx line (CPTI-000601) exhibits YFP expression that is indistinguishable from wild type Ubx expression in embryos and in imaginal discs [31]. While flies homozygous or hemizygous for the Ubx-YFP allele exhibit reduced viability, the morphological phenotypes are very weak indicating that Ubx function is substantially normal. For Hth, we selected a line, CPTI-000378, showing nuclear YFP expression corresponding to the endogenous *hth* pattern [32], [33]. Although CPTI-000378 is homozygous lethal, it is viable and phenotypically normal over *hth* ^C1^, a strong hypomorphic *hth* allele, indicating that the Hth protein trap provides substantial Hth function. For the ChIP-array analysis, we compared the specific signal derived from immunoprecipitation of chromatin from a YFP-protein trap line with anti-GFP/YFP antibody versus the control signal from chromatin taken from the isogenic wild-type progenitor immunoprecipitated with the same anti-GFP/YFP antibody. We used Drosophila 2.0 Affymetrix genome tiling arrays and performed three biological replicates for each sample. For both Ubx-YFP and Hth-YFP, genome-wide binding was assayed using chromatin samples from 0–16 hr embryos and 3^rd^ larval instar haltere imaginal discs; for Hth-YPF we also assayed binding in 3^rd^ larval instar wing imaginal disc chromatin. For each dataset we identified bound regions according to a False Discovery Rate (FDR) model using the TiMAT software (http://bdtnp.lbl.gov/TiMAT/TiMAT2/; summary of dataset analysis in Table S1). The data generated from imaginal disc chromatin shows improved signal-to-noise compared to that from embryo chromatin perhaps reflecting the benefit of using a restricted tissue where more cells share the same binding events rather than the heterogeneous cell mixture in whole embryos. For most of the analysis presented here we focus on the haltere data set.

### Analysis of Ubx binding

We used the haltere imaginal disc data to derive a set of direct Ubx targets. Haltere development represents a classic example of the role of homeotic genes in segment specification [34], [35]. In the wild type, the dorsal imaginal discs in the third thoracic (T3) segment express the Hox gene Ubx and develop into small rounded appendages, the halteres. Ubx is required for haltere specification since in the absence of Ubx function these discs produce wings, the appendages normally found on the second thoracic (T2) segment. Ubx is also sufficient for haltere specification versus wing since over-expression of Ubx in T2 discs converts the developmental program from wing to haltere [36], [37]. Specifying haltere versus wing involves the regulation of many developmental processes including the number of cells allocated to the imaginal primordia in the embryo, control of both cell division and growth as well as the regulation of pattern formation and differentiation [26], [38]–[41].

We find widespread Ubx binding across the genome in haltere chromatin. At a stringent 1% FDR threshold we identify 1,875 bound regions associated with 1,147 (Table S2). In the analysis that follows we mainly focus on the bound regions and corresponding genes identified at 1% FDR, though we do use less stringent FDR levels when comparing our ChIP profiles with other datasets. Supporting the view that we have identified *bona fide* Ubx binding regions in the *Drosophila* genome, we find that 96% of our high confidence Ubx bound regions are also associated with Ubx binding in an independent ChIP-array study performed by Slattery *et al.* (Personal Communication; Figure S1).

To link these bound regions with functional *Ubx* regulation we used available gene expression data. Since Ubx is solely responsible for the specification of haltere versus wing, genes differentially expressed between wing and haltere are either directly or indirectly downstream of Ubx. There are two sources of such genes currently available: first, there are a small number of genes (53) whose expression patterns, as assayed by in situ hybridisation or immunolabelling, differ between wing and haltere (Table S3). For five of these there is evidence that they are direct Ubx targets, for others the regulation may be either direct or indirect. We find that 28 (53%) of these genes are associated with Ubx binding at 1% FDR and 89% are bound at the less stringent 25% FDR,. Two of the five characterised direct targets are bound by Ubx at 1% FDR and all five are bound at 25% FDR. Second, three groups have used gene expression microarrays to identify genes differentially expressed between wing and haltere, either by directly comparing each tissue or comparing normal wing discs with those misexpressing Ubx [10], [11], [42]. Overall, we find 294 (20%) of the 1,488 Ubx-regulated genes identified in the in situ or microarray studies overlap with our list of genes associated with Ubx binding in haltere discs (Table S2). This highly significant (p = 0.0001) overlap strongly supports the view that at least 294 (26%) of the Ubx-bound genes we identify are likely to be direct Ubx-regulated targets.

The 26% overlap with Ubx-regulated genes is likely to be an under-estimate. First, there is little overlap between the three different gene expression studies with less than 1% overlap in the total of 1,605 genes identified (Figure S2). This indicates that the gene expression profiling is not close to providing a comprehensive listing of regulated genes. Second, the most recent and detailed analysis [42] concentrates on a restricted region of the disc (the pouch region) and, in addition, finds little overlap between Ubx-regulated genes at three different time-points again indicating that the list of regulated genes is likely to be far from complete.

**Figure 1:**
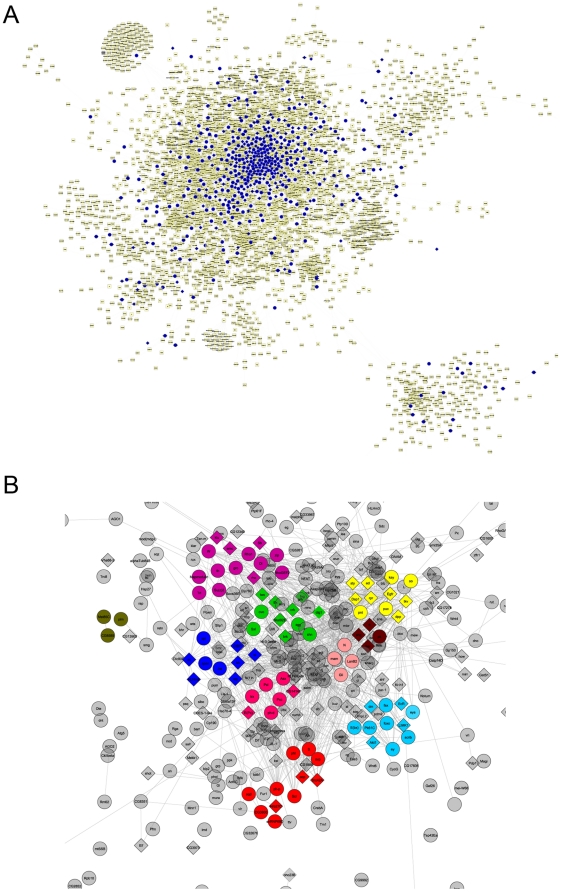
Ubx-bound genes are widely distributed across the *Drosophila* 20K gene network. (A) Ubx-bound genes (blue) are mapped onto the network visualised in Cytoscape [43]. (B) Ubx-bound genes (294 gene set as diamonds and remaining genes of the 1,147 set as circles) with selected subclusters coloured.

**Figure 2:**
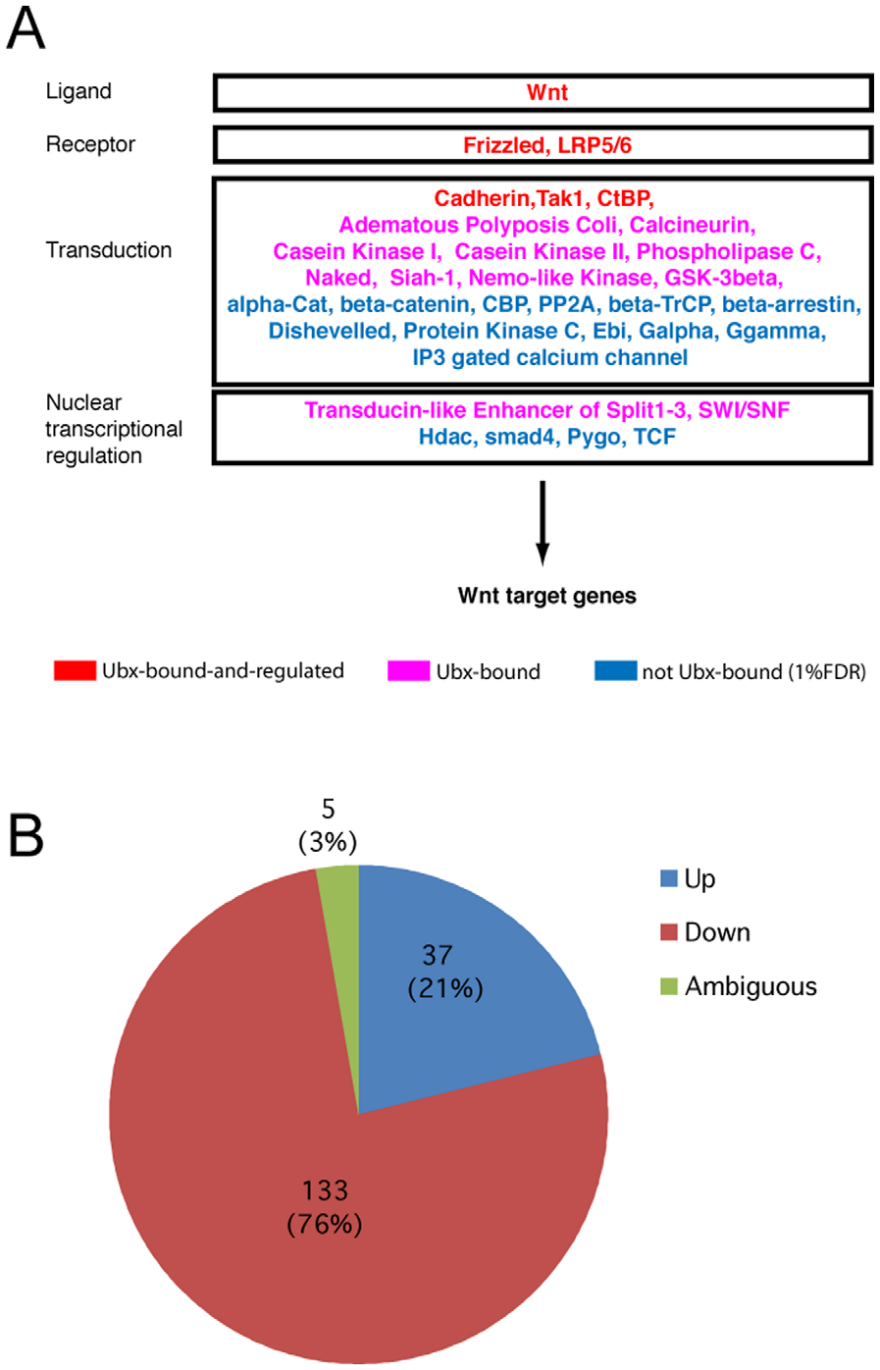
Features of Ubx-bound genes. (A) Wnt/wingless pathway components from Panther are listed and coloured according to presence of corresponding genes in: 294 gene set (Ubx-bound and supported by regulation; red), remaining genes of 1,147 Ubx-bound gene set (pink) and genes not in the 1% FDR Ubx-bound list (blue). (B) Genes from the 1,147 Ubx-bound gene set that overlap with differentially expressed genes from the Mohit et al. [10], Hersh et al. [11] and the larval genes from Pavlopoulos and Akam [42] classified according to direction of regulation by *Ubx*.

Plotting the 1,147 Ubx-bound genes (and the regulation validated subset of 294 genes) onto the Drosophila 20K gene network [43], reveals that they are spread broadly across the functional network indicating involvement in a wide range of processes (Figure 1). Out of 111 clusters in the entire network, we find 43 clusters (39%) associated with Ubx-bound genes. To determine the gene functions involved, we examined the GO biological process classifications associated with the 1,147 Ubx-bound and the 294 Ubx-bound-and-validated genes (Table 1). Genes associated with developmental processes are strongly over-represented together with highly relevant sub-classes such as ectoderm development. In support of previous studies indicating that Hox genes are likely to act at multiple levels in developmental pathways [26], [39], we find that enriched classes do not only represent higher level control functions (e.g. mRNA transcription regulation and signal transduction) but also the more basic morphogenetic functions (e.g. cell adhesion and cell motility). The more basic functions are represented by proteins such as the cadherins (Shotgun and Cadherin-N), other cell adhesion molecules (e.g. Neuroglian, Dally and Dally-like) and the cell death protein Reaper. Also, in line with studies showing the key roles of Ubx regulation of signalling pathways in haltere morphogenesis, we find over-representation of several signal transduction pathways including the Notch and Wnt-signalling pathways. As anticipated from the previous studies, within these pathways we find Ubx targets at multiple levels from ligands to receptors and effector mechanisms (Figure 2).

**Table 1:** Gene ontology and other function enrichments associated with Ubx-bound genes that have identified expression changes or the complete set bound by Ubx at 1% FDR.

	Ubx-bound (supported by expression)		All Ubx-bound Genes	
Biological Process	Genes	p	Genes	p
Developmental Processes	69	9.8E−22	176	1.2E−30
Neurogenesis	29	4.7E−11	67	9.8E−15
Ectoderm Development	29	1.2E−10	70	2.3E−15
mRNA Transcription	49	1.1E−09	144	2.7E−18
mRNA Transcription Regulation	41	2.6E−09	116	7.5E−17
Cell Communication	25	8.7E−09	56	2.1E−10
Signal Transduction	52	4.0E−08	154	1.9E−13
Cell Adhesion	18	7.1E−07	40	5.2E−08
Cell Adhesion-Mediated Signalling	13	8.4E−07	23	3.9E−06
Nucleoside and Nucleotide Metabolism	62	3.3E−05	198	5.3E−09
Cell Motility	13	7.6E−04	35	1.8E−07

Signalling Pathways				
Cadherin signalling	8	8.1E−04	20	1.4E−07
Wnt signalling	13	5.7E−04	34	2.7E−07
Presenilin Pathway	8	3.5E−03	19	2.0E−05

Looking at the effect of Ubx on the expression of genes in halteres or transformed wings suggests that Ubx may predominately act as a repressor of direct target genes in the haltere. Although the overall percentage of down-regulated genes at the larval stage in the differential expression datasets is 65%, we find a significantly stronger bias towards repression in the Ubx-bound genes (76%, p = 0.0004; Figure 2).

Interestingly, the full set of 1,147 Ubx-bound genes and the subset of 294 Ubx-bound-and-validated genes have very similar GO profiles (Table 1), supporting the view that many of the 1,147 genes identified at the stringent 1% FDR are likely to be functional Ubx targets. The overlap with genes identified in genetic screens for loci involved in imaginal disc development also strongly emphasises the specific functional relevance of the 1,147 Ubx-bound gene set: for example, of the 373 genes identified in a screen for genes implicated in wing vein formation [44], 111 are Ubx-bound in the haltere disc (p = 1.1E−37). This striking enrichment clearly demonstrates that the set of Ubx-bound genes are functionally important in aspects of imaginal disc development.

### Multiple-peak versus single-peak target genes

Scanning across the genome we find that Ubx binding occurs both as isolated peaks and also in concentrated domains of binding that contain multiple peaks. We separated the target genes into three sets; single-peak (305 genes), multiple-peak (323 genes) and unassigned (519 genes). While the length of single-peak genes is similar to the genome average (5.8 kb compared to the genome average of 5.6 kb), the multiple-peak genes are associated with much larger transcription units (average length 34 kb). Strikingly, the two assigned gene sets have very different functional signatures. While the single-peak genes show little GO class enrichment (only “Intracellular protein traffic” is significantly enriched), the multiple-peak genes display a set of significant GO enrichments similar to that of the full set of 1,147 Ubx-bound genes (Figure S3).

### Ubx binding and temporal developmental control

**Figure 3:**
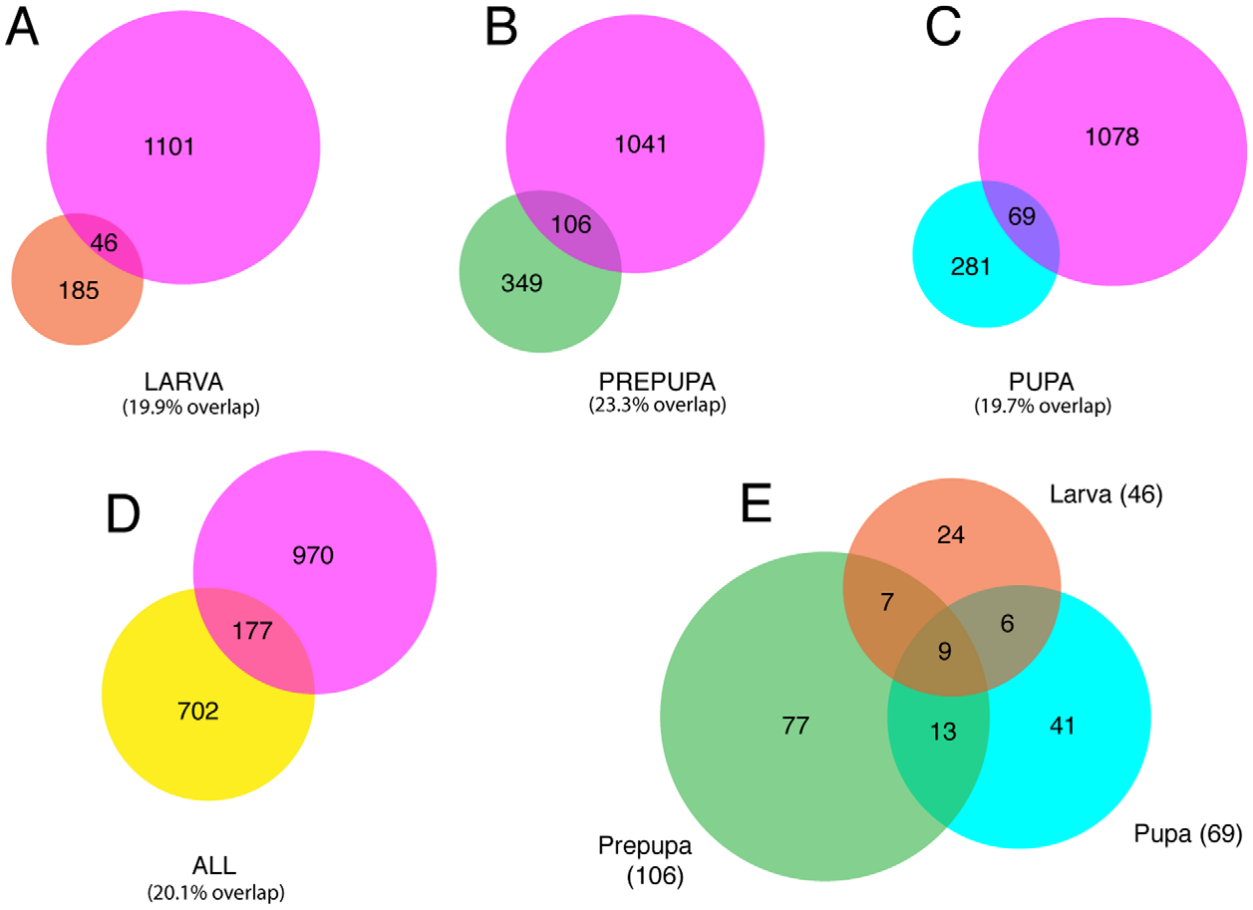
Temporal targets. (A–D) Overlaps between the 1,147 Ubx-bound gene set (purple) and the differentially expressed genes from Pavlopoulos and Akam [42] at the larval (brown), prepupal (green), pupal (teal) or combined (yellow) timepoints. (E) Overlaps between the Ubx-bound genes at the three different timepoints in the Pavlopoulos and Akam [42] data.

In the study by Pavlopoulos and Akam [42], Ubx-dependent differential gene expression was analysed at three time points encompassing approximately 20 hrs of development; late 3^rd^ instar larva, pre-pupa and early pupa. As indicated above, a striking conclusion of this study is that the sets of Ubx regulated genes are largely distinct at each time point. Since we analysed Ubx binding in haltere discs from 3^rd^ instar larvae, we examined whether there is a particular relationship between Ubx binding and the Ubx-regulated genes identified at this same stage. Interestingly, we find a very similar degree of overlap between Ubx-bound genes and Ubx-regulated genes at each of the three timepoints (Figure 3), suggesting that genes responding to Ubx during the pupal stage are already bound by Ubx at least 20 hrs earlier during the 3^rd^ larval instar. Thus it appears that Ubx binding is not necessarily associated with active gene regulation, but that it may set the context for future regulation, for example when a gene is subsequently activated via a signalling pathway.

### Analysis of Hth binding

**Figure 4:**
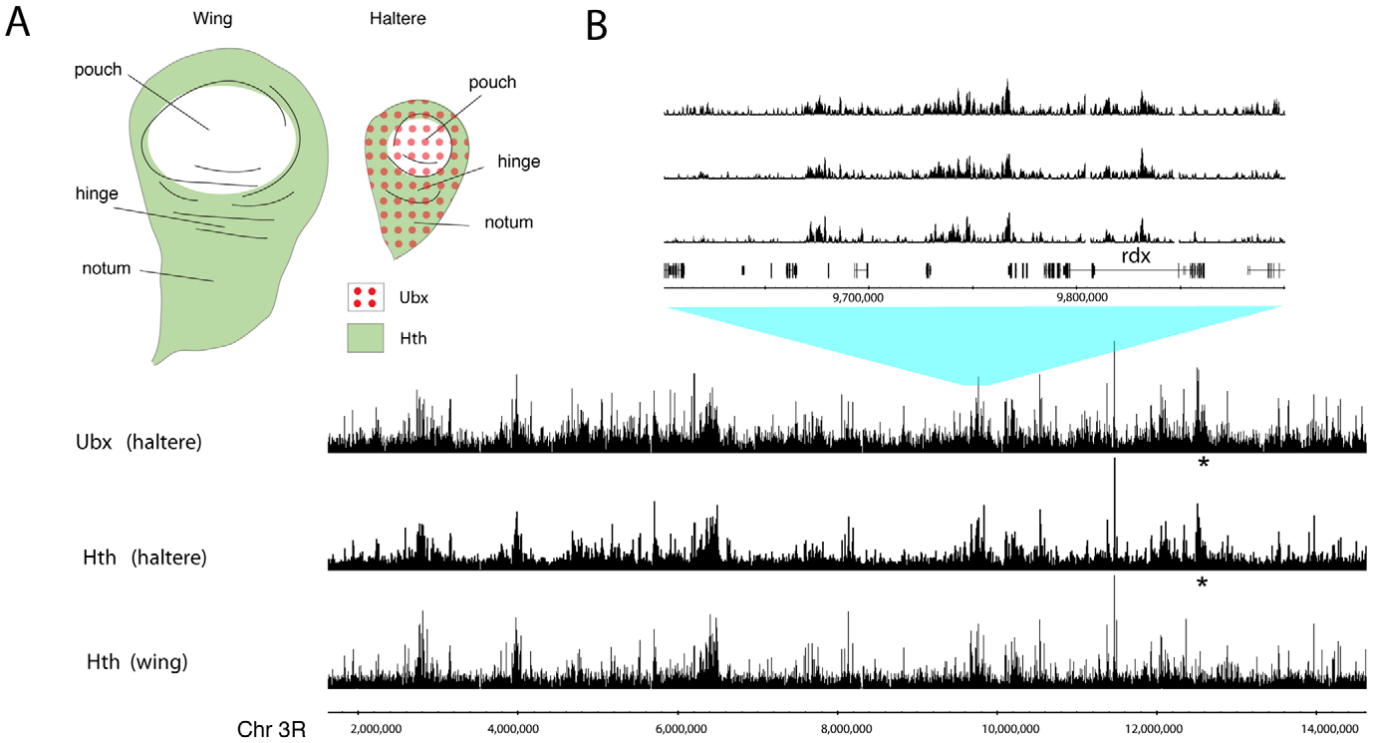
Comparison of Ubx and Hth binding profiles. (A) Schematic of Ubx and Hth expression in the wing and haltere discs. The wing disc pouch region gives rise to the wing blade and the haltere pouch region gives rise to the haltere capitellum. We use the term haltere hinge to encompass the pedicel and scabellum. (B) Log_2_ enrichment ratio profiles for Ubx and Hth on representative regions from chromosome 3R. The peaks at approx 12,500,000 (asterisk) present in the haltere profiles and absent in the wing are associated with the *Ubx* gene (see Figure 7).

Whereas Ubx is expressed widely in the haltere disc and functions in the pouch, hinge and notum to specify T3 segment identity, the Hox cofactor Hth shows more limited expression (Figure 4). Hth is expressed in the hinge and notum regions of the 3^rd^ instar haltere discs, where it functions in segment specification and also has a major role in the development of the proximo-distal axis [33], [45]–[47]. In the notum, Hth is required for the nuclear localization of Exd [33] and thus functions together with Ubx in specifying T3 development as exd^-^ clones transform the T3 notum to T2 [48]. In the pouch region, Hth is not expressed and neither Hth nor Exd are required for the Ubx-dependent specification of wing blade versus haltere capitellum [49]. This is illustrated by the regulation of spalt major (salm), which is expressed in the wing pouch but is repressed in the haltere pouch by Ubx independently of hth or exd. Analysis of the salm pouch-specific regulatory element revealed a tandem array of Ubx binding sites suggesting that Ubx multimerisation might obviate the requirement for Hox cofactor binding at specific target genes [21].

Strikingly, we find that in haltere chromatin the Hth genomic binding profile is very similar to the Ubx profile (Figure 4, Table 2 and Figure S4) with over 97% of Ubx-associated genes also associated with Hth. At higher resolution, over 99% of Ubx-bound regions are associated with Hth (p = 0.001). There could be several possible reasons for this close association of Ubx and Hth binding. It could reflect clustering of Ubx and Hth binding sites in keeping with their function in a Hox/Hox-cofactor complex. Alternatively, it may reflect a strong influence of chromatin accessibility on the binding profile coupled with low-specificity widespread binding of both homeodomain proteins. These explanations are not mutually exclusive and the similarity of the binding profiles could result from a mixture of the two.

**Table 2:** Percentage overlap between ChIP profiles in terms of bound regions and unique genes when peaks at 1% FDR are compared with 25% FDR datasets.

	Bound Regions (25% FDR)			Unique Genes (25% FDR)		
	Ubx haltere	Hth haltere	Hth wing	Ubx haltere	Hth haltere	Hth wing
Ubx haltere (1% FDR)	100	99	83	100	97	89
Hth haltere (1% FDR)	67	100	62	81	100	79
Hth wing (1% FDR)	82	99	100	93	98	100

### Investigating similarity of the Ubx and Hth binding profiles

**Figure 5:**
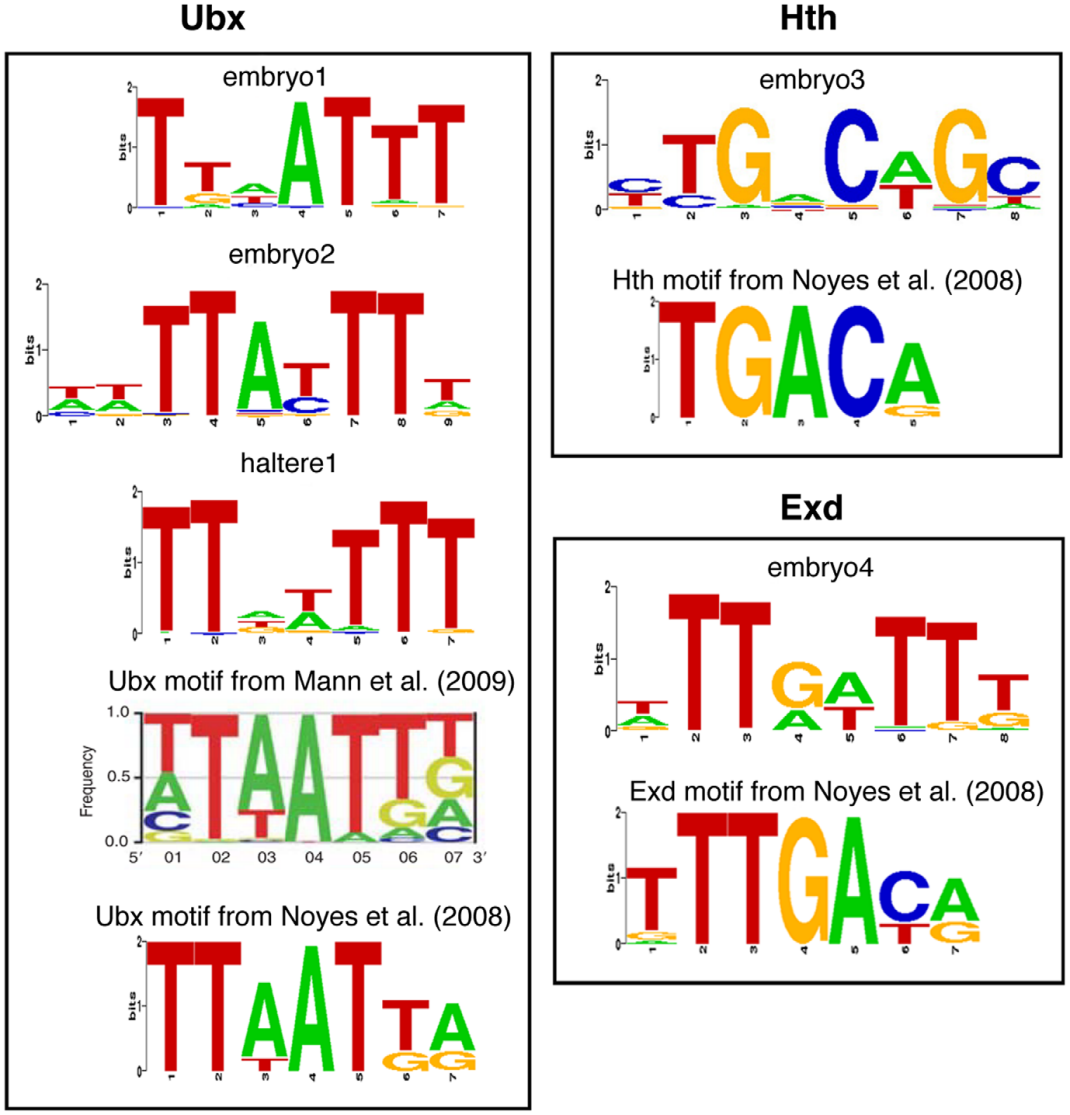
Sequence motifs identified. Enriched motifs derived from the Ubx and Hth ChIP-array data are compared to previously identified motifs from Mann et al. [5] and Noyes et al. [52].

In order to understand the binding specificity of Ubx and Hth we looked for enriched sequence motifs underlying the binding peaks. For Ubx, we used the top 300 binding peaks and performed motif discovery analysis using nestedMica [50] for the embryo and haltere data separately. We found motifs containing a TAAT-like core site which are similar to the Ubx or Hox binding motifs identified from in vitro studies [51], [52] (Figure 5). The consensus sequence of the embryo1 motif (TTAATTT) is the same as the Ubx motif derived from in vivo validated Ubx binding sites [5]. In the case of Hth, motif searching with peaks bound only by Hth identified a motif (CTGACAG) that is similar to a Hth motif (TGACA) identified in a bacterial one-hybrid screen [52]. We also found a potential EXD motif that contains a TGAT core site [52], [53]. Motif searching on peaks bound by both Ubx and Hth did not identify enriched motifs resembling any of the in vitro defined motifs, in particular, we did not find motifs corresponding to the proposed cooperative Hox/Pbx TGATNNAT[g/t][g/a] site or to any of the proposed Ubx/Exd preferential sites TGATTTAT,TGATTTATTT, or ATGATTTATGG [5], [23], [54], [55]. In addition, we directly searched for matches to TGATNNAT[g/t][g/a] and TGATTTAT/TGATTTATTT/ATGATTTATGG in both the top 1000 embryo Ubx binding peaks and the 1875 haltere binding peaks but found none of these motifs significantly enriched in either dataset. Overall, our data suggest some relevance of previously known motifs for the in vivo genomic sites we identify, however, these frequently occurring short motifs do not explain the binding profiles we observe. Other enriched motifs represent candidates for potential cofactor binding sites and we note good matches to the characterised sites for Pho, Brk and Dref in motifs discovered from the embryo data (Figure S5).

Collaborative binding of Ubx and Hth could provide an explanation for the similarity between binding profiles, however we believe this is not likely. First, as mentioned above, Hth is not detectably expressed in the cells of the haltere pouch where Ubx is required to specify haltere fate. Second, we examined the binding profile of Hth in the wing imaginal disc and find that it is very similar to the haltere disc profile (Table 2). There is very little Ubx expression in the wing imaginal disc [31], indeed most of the cells entirely lack any Hox protein expression [56], thus the binding profile of Hth in the wing disc cannot reflect Hox/Hox-cofactor collaboration.

**Figure 6:**
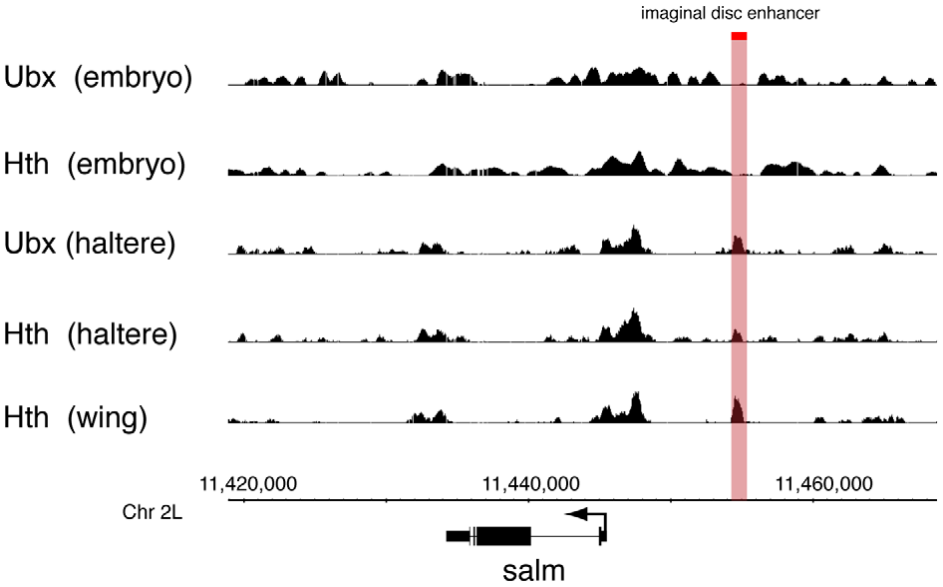
Ubx and Hth binding at *salm.* The red vertical indicates the imaginal disc enhancer identified by Galant et al. [21].

Focusing on one of the best characterised Ubx target genes in the haltere disc, the salm gene, we find extensive correspondence between Ubx and Hth binding (Figure 6). In haltere disc chromatin both Ubx and Hth bind to the disc regulatory element identified by Galant et al. [21]. In a reporter assay this element drives expression in the wing disc pouch but *Ubx* directly represses it in the haltere disc. Since Hth is not expressed in the haltere disc pouch, Ubx regulation of the element is clearly independent of Hth. However, our data show Hth clearly bound at this element in the haltere despite having no known function. We examined whether hth mutant clones have any effect on salm expression outside the pouch, but found no effects (data not shown). We conclude that the binding of Hth to the salm disc regulatory element may be non-functional.

We note that Hth is not bound at this region in the embryo but is bound in both the wing and the haltere discs, an observation consistent with the Hth binding reflecting developmentally-regulated chromatin accessibility (Figure 6). In general, the genome-wide binding profiles of Ubx and Hth in embryo chromatin appear quite different from the imaginal disc profiles, suggesting that target selection by these proteins undergoes a widespread developmental reorganisation.

### Role of chromatin: Polycomb silencing excludes binding of Ubx and Hth

**Figure 7:**
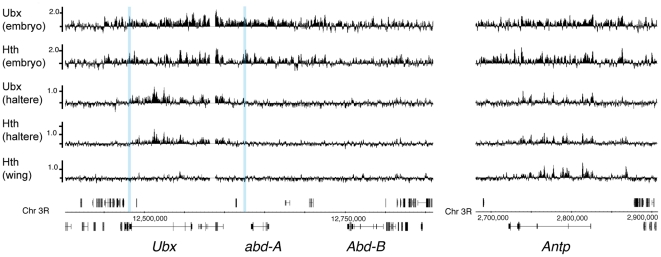
Ubx and Hth binding at the Bithorax complex and *Antp*. The embryo chromatin represents a heterogenous mixture of epigenetic ON and OFF states at the Bithorax genes and at *Antp* imparted by the Pc/Trx machinery. In the haltere disc chromatin *Ubx* and *Antp* are epigenetically ON, *abd-A* and *Abd-B* are OFF. In the wing disc *Ubx*, *abd-A* and *Abd-B* are OFF, whereas Antp is ON. The Bithorax Complex is on the left. The blue verticals represent the position of insulator component binding sites (CP190 and CTCF; [63]). The *Antp* locus is on the right.

To explore the possible link between chromatin and the observed profiles of Ubx and Hth binding, we examined the Bithorax complex since the epigenetic chromatin state in this region has been characterised in imaginal discs [29], [57]–[60]. The Bithorax complex contains the three Hox genes Ubx, abd-A and Abd-B [35], [61]. In the haltere disc, Ubx is ON whereas abd-A and Abd-B are OFF due to heritable silencing by the Polycomb (Pc) machinery. In haltere disc chromatin, we find that Ubx and Hth are bound at multiple peaks in a large domain spanning the Ubx transcription unit and associated 5′ regulatory region (Figure 7). This domain is bounded by insulator sites, corresponding to the regulatory domain architecture of the Bithorax complex [62], [63]. In contrast, the silenced genes, abd-A and Abd-B, show virtually no evidence of Ubx or Hth binding suggesting that Pc silencing may block access of Ubx and Hth to these regions. This situation does not simply reflect the distribution of Ubx and Hth binding sites as Ubx and Hth are bound across the whole Bithorax complex in the embryo. The embryo chromatin represents a heterogeneous mixture of cells with each Bithorax complex gene in an ON state in some cells in the embryo. The relevance of the epigenetic activity state is supported by the analysis of Hth binding in the wing imaginal disc. Here Ubx is predominantly silenced [59] and, in contrast to the domain of Hth binding over the Ubx gene seen in the haltere disc, we find little binding over the Ubx gene in the wing disc. This is further supported by the analysis of binding at the Antennapedia (Antp) Hox gene where we find Ubx and Hth binding at multiple peaks across the gene in haltere disc chromatin and also a similar binding profile for Hth in the wing disc (Figure 7). This is interesting as both these discs, in the T2 and T3 segments respectively, are derived from the region of the embryo where Antp is epigenetically ON as Antp is expressed posteriorly from T1 [64], [65].

Although Antp should be epigenetically ON in both wing and haltere discs it is only detectably expressed in a few cells in these discs in the 3^rd^ larval instar [56]. This separates the heritable epigenetic state of the gene from its state of transcriptional activity and suggests that binding of Ubx and Hth may be associated with the ON chromatin state rather than with transcriptional activity per se.

**Figure 8:**
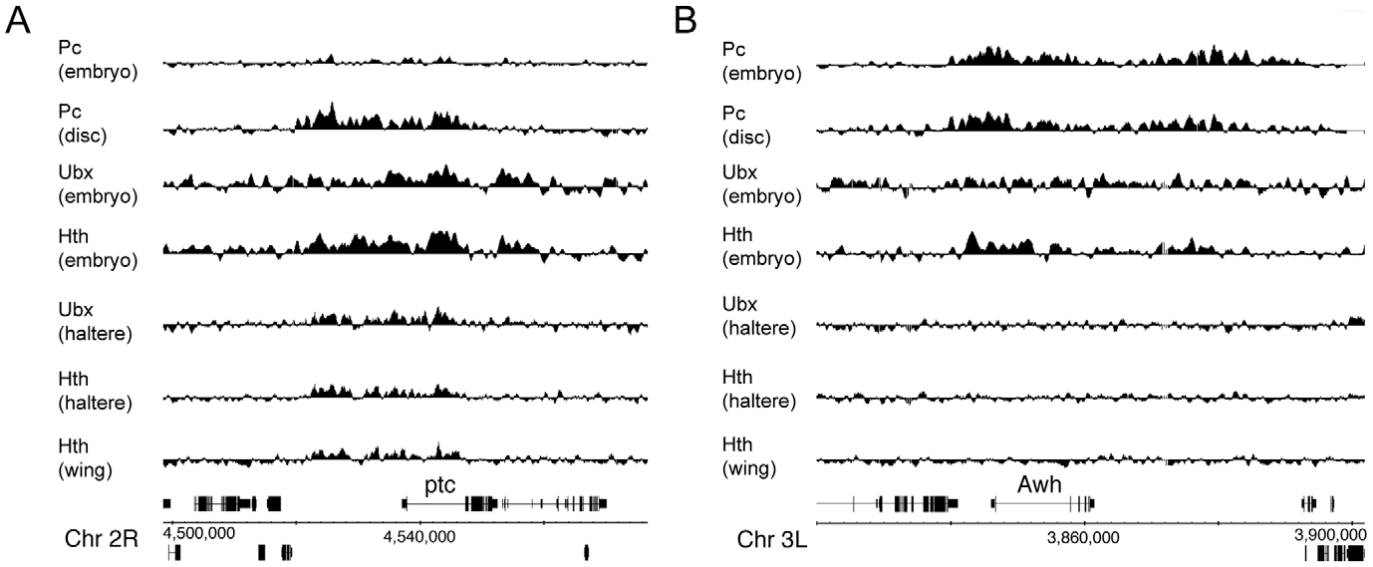
Ubx and Hth binding at Pc target genes. Examples of Pc target genes that are active or repressed in imaginal discs. (A) *ptc* is expressed in wing and haltere disc and is associated with Ubx and Hth binding. (B) *Awh* is likely to be predominantly silenced in imaginal discs and is bound by Ubx and Hth in the embryo but not in the imaginal discs. The Pc binding data from embryo and from T3 (haltere and leg 3) imaginal discs is from Kwong et al. [29].

Restriction of Ubx and Hth binding to Pc target genes in the ON state may not only be a feature of the Hox complexes. We examined several Pc target genes that are expressed in imaginal discs (e.g. engrailed, hedgehog, hth, patched and vestigial) and found that they are associated with significant Ubx and Hth binding (Figure 8). Identifying genes that are definitively in the silenced OFF state in imaginal discs is more difficult, however two candidates are Arrowhead and tinman. Arrowhead is expressed in very few imaginal disc cells and general ectopic expression in imaginal disc causes cell death [66]–[68]. Although both these genes bind Ubx and Hth in embryo chromatin, they do not bind in the imaginal disc chromatin where they are likely to be Pc silenced (Figure 8). Taken together, these observations support the view that aspects of Ubx and Hth binding reflect the accessibility of particular chromatin regions during development rather than being solely driven by underlying DNA sequence motifs.

## Discussion

Twenty years ago, in the pre-genomic era, our attempt to identify Ubx target genes using ChIP resulted in the characterisation of 2 Ubx targets [9]. Here, using ChIP-array, we identify 1,147 genes associated with Ubx binding in the haltere imaginal disc and for 294 of these corroborating RNA expression data suggests that Ubx regulates their transcription [10], [11], [42]. These genes show striking enrichment for functions associated with developmental processes and with signalling pathways such as the Wnt and Notch pathways. Although transcription factors and signalling molecules are well represented, indicating Hox regulation of high-level control processes, there are also target genes representing more basic functions such as cell adhesion, cell motility and apoptosis. This fits well with earlier analyses indicating multi-level control of developmental processes by Hox genes [26], [69].

Our data support previous studies indicating Ubx regulation of the wg (Wnt), dpp (TGFβ) and EGF pathways [26], [38], [40], [41], [70] and provide further evidence for direct regulation of several genes in these pathways. In addition, we provide evidence for direct Ubx regulation of genes involved in several other pathways including the Notch pathway (represented by *Delta, E(spl) complex, fringe, Notch* and *numb*), the fat pathway (represented by *dachsous, discs overgrown, expanded, fat* and *four-jointed*), the hedgehog pathway (represented by *cubitus interruptus, discs overgrown, gilgamesh, hedgehog, patched* and *shaggy*) and the ecdysone pathway (represented by *Ecdysone receptor, ecdysoneless, L-lactate dehydrogenase* and several *Ecdysone-induced* genes).

A feature of our genome-wide binding data is the very close similarity between the binding profiles of the Hox protein Ubx and the cofactor Hth in the haltere disc: a surprising observation for several reasons. First, these two homeoproteins bind distinct sequence motifs in vitro [52]. Second, they represent binding events in different populations of cells, since Ubx is expressed over the whole disc [31] while Hth is expressed in the proximal regions of the disc, including the presumptive hinge and notum, but not in the pouch region [33]. The major Ubx-dependent transformation between wing blade and haltere capitellum does not require the Exd/Hth cofactors so the regulatory elements responsible for Ubx-target gene regulation in this region, such as the characterised element at salm, are expected to bind Ubx but not Hth [21]. Third, although much of Hth function may be associated with its role as a Hox cofactor there is evidence for additional Hox-independent Hth functions [71]. For example, in imaginal discs hth is associated with the regulation of proximo-distal axis development [33], [45]–[47], which might be expected to involve different target genes than those involved in segment specification by Ubx. Although almost all Ubx-bound regions are also associated with Hth (Table 2), our data do not rule out bona fide subsets of sites associated with Ubx or Hth alone and we note that there is a subset (33%) of Hth-bound regions that are not associated with strong Ubx binding. Nevertheless, the predominant feature that we emphasise here is the similarity between Ubx and Hth binding profiles. Our observations are reminiscent of the studies of transcription factor binding in the Drosophila blastoderm where disparate transcription factors show similar binding profiles and this has been interpreted to represent a strong influence of chromatin accessibility on transcription factor binding [72], [73]. Most transcription factors recognise small degenerate motifs and if single occurrences of these motifs in accessible chromatin give sufficient occupancy to generate a ChIP signal, then even short blocks of accessible chromatin may be seen to bind large numbers of different DNA binding proteins. For example, Ubx binds the sequence TAAT and in random sequence this motif would be present every 128 bp on average and so the release/remodelling of a single nucleosome generating 150 bp or so of accessible DNA is quite likely to reveal a Ubx site. An alternative view is that stable binding is only observed at sites where Ubx can bind in association with cofactors such as Exd/Hth. A consensus site (TGATNNAT[g/t][g/a]) has been derived for Hox/Exd binding [54], [55] however we do not find clear matches to this motif in our analysis of sequence motifs enriched at binding sites and direct searching did not reveal enrichment. At the resolution of ChIP analysis, the combination of binding at degenerate small motifs and a strong influence of chromatin structure on accessibility would generate very similar binding profiles for different transcription factors binding distinct motifs. In this situation only a proportion of the potential binding sites in the genome would be accessible and bound in any cell. In different tissues, with distinct chromatin accessibility profiles, different binding sites would be occupied. This idea fits with the very different binding profiles for Ubx and Hth we observe comparing embryo versus haltere disc chromatin. This situation contrasts with our analyses of the multi-zinc finger insulator proteins Su(Hw) and CTCF which have long binding motifs, where sequence motif matches in the genome are good predictors of binding and where binding is very similar between tissues [28], [62], [74].

By profiling binding in a specific tissue where we know the chromatin states of particular genes, we can link Ubx/Hth binding with chromatin state. We find that the Bithorax complex genes abd-A and Abd-B which are silenced in the haltere disc and packaged by the Pc machinery into a repressive chromatin domain, are not accessible for binding by Ubx and Hth. In contrast, the Ubx gene is active and accessible for binding Ubx and Hth. The boundary between the accessible Ubx region and the inaccessible abd-A/Abd-B region corresponds to an insulator site, an observation that supports the domain model of the Bithorax complex where regulatory domains, separated by insulators/boundaries, can independently be set to different chromatin states by the Pc machinery [62], [75]. Our data provide strong support for the idea that chromatin state controls access of transcription factors to their binding sites. Specifically, we show this for a particular chromatin state, the Pc silenced state, but the overall similarity of the Ubx and Hth binding profiles suggests that, in general, chromatin state may exert a strong influence on transcription factor binding.

Attempts to probe the DNA accessibility within Pc repressed domains have given conflicting results. Although Pc repressed chromatin does not affect the accessibility of restriction enzymes [76] it does block the activities of the Gal4 activator, the FLP recombinase, and two forms of T7RNAP [77], [78]. Our studies indicate a profound block to transcription factor binding across the whole repressed domain. However, the repressed domain is not impervious to components of the transcriptional machinery [79], [80] and the Abd-B promoter within the repressed domain in haltere discs is associated with stalled RNA polymerase [81].

The inability of Hth to bind within Pc repressed regions contrasts with evidence in muscle differentiation that Pbx and Meis proteins, the vertebrate orthologues of Exd and Hth, may function as “pioneer factors”, acting at an early stage in gene activation by penetrating repressed chromatin [82]. Our data do not support this idea as they suggest that Pc repression in particular, and chromatin state in general, limits Hth access to DNA.

While chromatin accessibility may go a long way toward explaining the ChIP binding profiles, the link between Ubx binding and transcriptional regulation remains unclear. For example, does the transient binding of Ubx to accessible low affinity sites affect target gene transcription or does Ubx need to assemble into a stable complex together with cofactors in order to regulate transcription? Either way, the role of chromatin accessibility would enable Hox proteins to act as modulators of existing gene regulatory programs which fits with the evolutionary role of Hox genes as modulators of segmental morphology [20]. In addition, if Hox proteins act on a background of accessible regulatory elements that differs according to cell state, this would provide a simple mechanism for Hox proteins to regulate appropriate target genes in different tissues and developmental stages.

## Materials and Methods

### Fly stocks and antibodies

The transgenic Ubx-YFP (CPTI-000601) and Hth-YFP (CPTI-000378) FlyProt protein trap lines were generated via a transposon-based exon-trapping screen [30]; details of these lines are available from http://www.flyprot.org/. The Ubx-YFP line has reduced viability; 31% of homozygotes survive to adulthood. The Hth-YFP line CPTI-000378 is homozygous lethal but the protein trap is viable over *hth* ^C1^, a strong *hth* hypomorph [83]. Wild-type flies used were the w^1118^ host stock used to generate the protein traps. A rabbit anti-GFP antibody [84] was used in all ChIP assays.

### ChIP

Chromatin from 0–16 h (after egg laying) old embryos was isolated as described previously [85]. For the preparation of chromatin from T2 wing and T3 haltere imaginal discs, late 3rd instar larvae were used. Discs were dissected out in PBS containing protease inhibitors then snap-frozen in liquid nitrogen and stored at −80°C. Chromatin was prepared from approximately 150 discs. The discs were homogenized in 20 µl cell lysis buffer (5 mM PIPES pH 8, 85 mM KCl, 0.5% Nonidet P-40) containing protease inhibitors using a motor driven small plastic pestle. 300 µl nuclear lysis buffer (50 mM Tris.HCl pH 8.1, 10 mM EDTA.Na_2_, 1% SDS) containing protease inhibitors were added to the chromatin extract and incubated for 20 min at room temperature. After the incubation, the extract was sonicated using a Bioruptor (Diagenode) at high setting for 4 min 15 sec. The sonicated chromatin was then flash frozen in liquid nitrogen and stored at −80°C.

Chromatin immunopurification was performed as described previously [85]. In all ChIP experiments, the specific IPs used chromatin from Hth-YFP and Ubx-YFP fly lines and the control IP used w^1118^ chromatin. Chromatin was incubated with anti-GFP (1 µl of 0.1 mg/ml affinity-purified antibody) overnight at 4°C. The ChIP wash conditions were 5 min with each buffer; once with low salt buffer (0.1% SDS, 1% Triton X100, 2 mM EDTA.Na_2_ pH 8, 20 mMTris.HCl, pH 8, 150 mM NaCl), high salt buffer (0.1% SDS, 1% Triton X100, 2 mM EDTA.Na_2_ pH 8, 20 mMTris.HCl, pH 8, 500 mM NaCl), LiCI buffer (0.25 M LiCl, 1% NP 40, 1% NaDeoxycholate, 1 mM EDTA.Na_2_, pH 8, 10 mM Tris.HCl, pH 8), and twice with TE (1 mM EDTA.Na_2_, pH 8, 10 mM Tris.HCl, pH 8). Chromatin was incubated at 67°C for 4 hours to reverse cross-linking, and DNA purified using PCR purification columns (Qiagen).

### Microarray analysis

Three biological replicates were used for each condition and enrichment profiles were generated by comparison of specific and control ChIP DNA samples. For the embryo samples, in order to obtain sufficient DNA (7.5 µg) for microarray analysis, 10–20 ng of ChIP and control DNA samples were amplified using Ligation-mediated PCR as described previously [86]. For wing or haltere disc chromatin, 0.6 ng was amplified using the GenomePlex Single Cell Whole Genome Amplification Kit (Sigma-Aldrich). For subsequent fragmentation using the Affymetrix protocol the original amplification protocol was modified by adding 2.3 µl of 10 mM dUTPs in the PCR master mix (total volume per reaction: 61 µl). The amplified DNAs were then purified, fragmented, TdT labelled and hybridized to the Affymetrix Drosophila genome Tiling Array 2.0 according to Affymetrix Chromatin Immunoprecipitation Assay Protocol (http://www.affymetrix.com/support/technical/manuals.affx). The ChIP-array data have been submitted to GEO under accession number GSE23864 and all data is MIAME compliant as detailed on the MGED Society website http://www.mged.org/Workgroups/MIAME/miame.html.

### Affymetrix array data processing

Affymetrix CEL files were processed using TiMAT (http://bdtnp.lbl.gov/TiMAT/TiMAT2). All analyses were based on Release 5 of Drosophila melanogaster genome. All the replicates were median scaled and quantile normalized against each other with CelProcessor using default settings. The log (base2) binding ratios were calculated by comparing specific IPs and control IPs (log (mean specific IP/mean control IP)). These ratios were then smoothed using a sliding window (675 bp) of trimmed means. The .sgr files, containing information about the enrichment signals were generated by ScanChip. The binding peaks were determined by the peak finding algorithm provided in the TiMAT package. Binding profiles were visualized with the Integrated Genome Browser (IGB) browser [87]. The .sgr files are provided as Datasets S1, S2, S3, S4, S5.

### Gene assignment

For each significant bound-region, surrounding target genes (FlyBase genes from UCSC database) were assigned to the bound-region. A gene was assigned to a bound-region if it directly overlapped with the region, otherwise the closest gene was assigned to the region. To determine the closest gene, the genomic distance between the centre of the bound-region and the end of each annotated gene 3′ or 5′ to the peak was used.

### GO enrichment analysis

Genes were functionally classified with Gene Ontology terms using the PANTHER 6.1 (Protein ANalysis THrough Evolutionary Relationships) Classification System [88]. Over- or under-representation of the GO terms was statistically determined using the binomial test and p-values corrected for multiple testing using the Bonferroni method in the PANTHER system. A corrected p-value better than 0.05 was regarded as significant.

### Monte Carlo simulation method

A random sampling approach was used to test the significance of overlaps between two gene lists. Two sets of genes were randomly generated from all genes in the whole *Drosophila* genome and the proportion of overlapping genes between the two gene sets was calculated. For testing the significance of down-regulated Ubx targets, 175 genes were randomly selected from the initial dataset (884 non redundant larval genes from the three genome-wide expression studies) and the proportion of down-regulated genes was calculated. This process was repeated 10,000 times and a p-value was calculated based on the number of iterations in which the number of overlapping genes is equal or more than observed overlap.

### Single- and multiple-peak gene classification

Ubx target genes (1% FDR) were classified into different classes using stringent criteria. A gene was defined as a single-peak gene if there is only one 10% FDR peak and no other peak (up to 25% FDR) associated with the gene. A gene was defined as a multiple-peak gene if there are at least four 10% FDR peaks associated with it. The genes that did not fit into the above criteria were classed as unassigned.

### Motif discovery

Searching for over-represented sequence motifs underlying Ubx/Hth binding regions used selected peaks as input to the nestedMICA algorithm [50] and default settings. All search sequences were 400 nt long and extracted around the peak centre positions. Motif widths were set from 6 to 25 bases. Statistical over-representation of motifs was determined by comparing the set of all Ubx/Hth peak sequences to 1,000 sets of random sequences of the same length drawn from the *Drosophila* genome. A Z-score was derived from the numbers of motifs observed in real peaks versus the occurrences for the 1,000 random sets. Motifs were visually inspected with MotifExplorer (https://www.sanger.ac.uk/Software/analysis/nmica/mxt.shtml) and statistically significant (Z-score>3) motifs with high information content were identified. To classify regions bound by both Ubx and Hth or Hth-only, we compared 10% FDR enriched regions bound by Ubx and Hth. To identify motifs underlying the regions from the two groups, we performed motif searches separately using regions bound by Ubx+Hth (276) and regions bound by Hth-only (500).

### Statistical co-occurrence analysis

The significance of Ubx and Hth co-localization at the peak and gene levels was assessed by permutation testing with the default settings in the Cooccur package [89].

## Supporting Information

Figure S1 Comparative analysis with Slattery et al. data. Comparison of our data with Slattery et al. (personal communication) using data from both groups processed using TiMAT. (A) Number of bound regions across the genome and unique genes associated with bound regions for each of the proteins in haltere chromatin. Asterisk indicates that 5% FDR was used for this dataset. (B) Overlap analysis comparing the bound regions/genes identified in one dataset at high stringency with the bound regions/genes from the other dataset at lower stringency (25% FDR). Overlap is defined as at least 100 bp overlap between two bound regions. This analysis reveals considerable overlap in the data sets and we note, in particular, that 96% of the bound regions at 1% FDR in our data are also found in the Slattery et al. data at 25% FDR. (C) Correlation of windowed log2ratio scores along the whole genome for Ubx in haltere chromatin. (D) Correlation of windowed log2ratio scores along the whole genome for Hth in haltere chromatin.
(0.75 MB TIF)

Figure S2 Overlap of differentially expressed genes identified in microarray experiments. Data from Hersh et al. [11], Mohit et al. [10] and combined timepoints from Pavlopoulos and Akam [42].
(0.23 MB TIF)

Figure S3 GO analysis of genes associated with multiple or single Ubx peaks. Red asterisks indicate significant over- or under-representation (p<0.05 Bonferroni corrected). Up arrows indicate over-representation, down arrows indicate under-representation.
(0.46 MB TIF)

Figure S4 Hth versus Ubx binding: correlation analysis. Correlation of windowed log_2_ratio scores along the whole genome. (A) shows the correlation of the binding profiles of Hth versus Ubx in the haltere disc. In general, the genome-wide binding profiles of the two transcription factors are very similar (r = 0.65) in the haltere disc. (B) shows the correlation of the binding profiles of Hth versus Ubx in the embryo. (C) shows the correlation of the binding profiles of Hth in the wing disc versus Hth in the haltere disc.
(0.59 MB TIF)

Figure S5 Candidate cofactor motifs. Enriched motifs derived from the Ubx and Hth ChIP-array data are compared to known motifs from the *Drosophila* Curated Transcription Factor Motifs database (http://www.bioinf.manchester.ac.uk/bergman/data/motifs/).
(0.78 MB TIF)

Table S1 Number of bound regions across the genome and unique genes associated with bound regions for each of the proteins in the indicated chromatin source at a range of false discovery rates. For analysis of Ubx target genes the 1181 genes at 1% FDR in haltere disc chromatin were used however the histone gene repeats were removed giving a total of 1147 genes (see Table S2). Comparison of numbers of bound regions or gene sets across different chromatin sources is difficult due to signal/noise differences and consequent threshold effects. For a direct comparison of Hth and Ubx targets see Table 2.
(0.03 MB DOC)

Table S2 Ubx-bound genes (1% FDR haltere data).
(0.40 MB XLS)

Table S3 Ubx-regulated genes identified by non-microarray approaches.
(0.03 MB XLS)

Data Sets S1 Windowed enrichment ratios (log2Ratios) for Ubx ChIP on haltere imaginal disc chromatin (.sgr format).
(9.5 MB TXT)

Data Sets S2 Windowed enrichment ratios (log2Ratios) for Ubx ChIP on 0-16 hr embryo chromatin (.sgr format).
(9.4 MB TXT)

Data Sets S3 Windowed enrichment ratios (log2Ratios) for Hth ChIP on haltere imaginal disc chromatin (.sgr format).
(9.5 MB TXT)

Data Sets S4 Windowed enrichment ratios (log2Ratios) for Hth ChIP on wing imaginal disc chromatin (.sgr format).
(9.5 MB TXT)

Data Sets S5 Windowed enrichment ratios (log2Ratios) for Hth ChIP on 0-16 hr embryo chromatin (.sgr format).
(9.4 MB TXT)
