# Supplementary figures and images for: Genome-Wide Analysis of the Binding of the Hox Protein Ultrabithorax and the Hox Cofactor Homothorax in Drosophila
Source: PLoS One. 2011 Apr 5;6(4):e14778. doi: 10.1371/journal.pone.0014778 (PMC3071696; doi:10.1371/journal.pone.0014778)

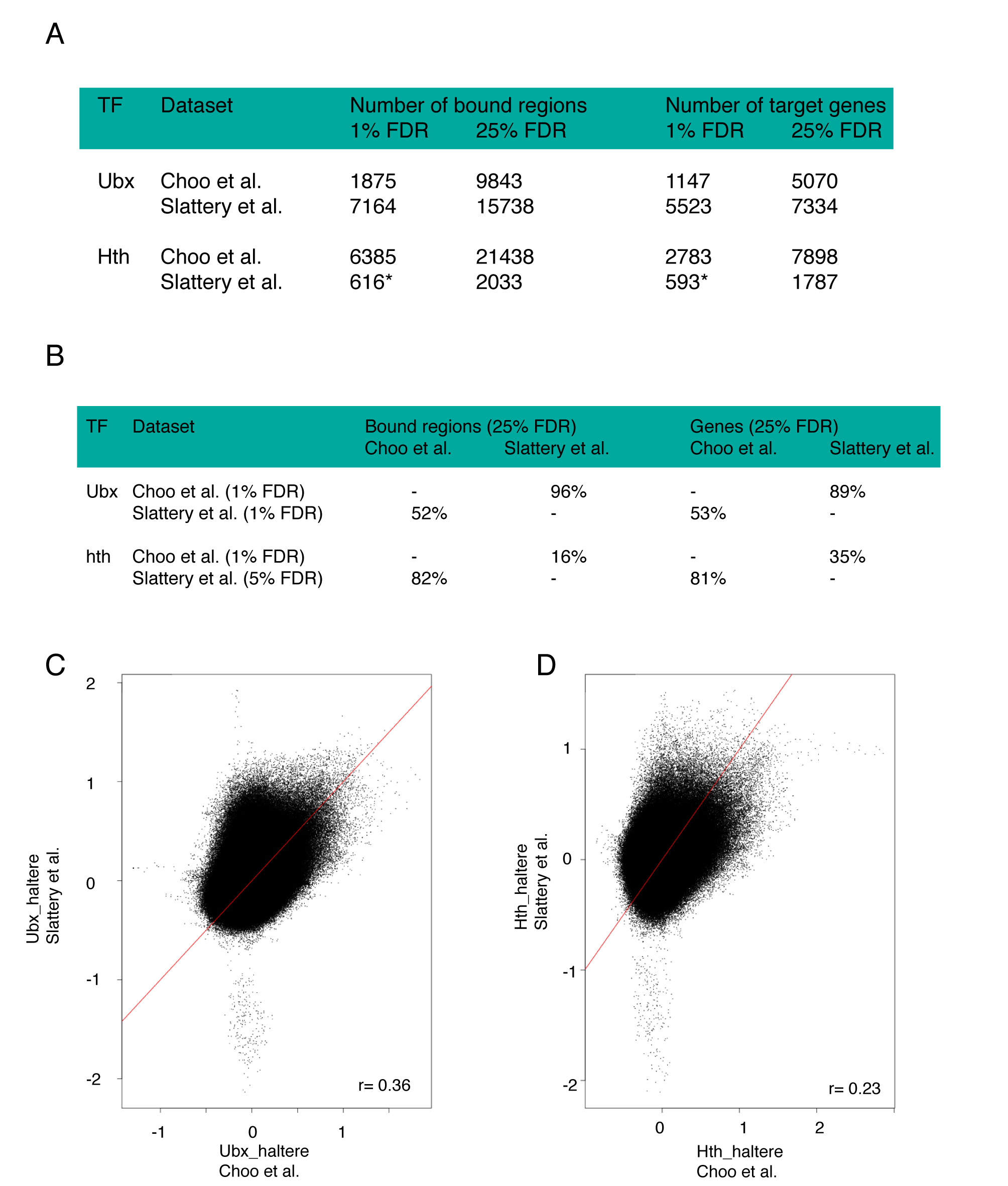

Supplement: Figure S1 — Comparative analysis with Slattery et al. data. Comparison of our data with Slattery et al. (personal communication) using data from both groups processed using TiMAT. (A) Number of bound regions across the genome and unique genes associated with bound regions for each of the proteins in haltere chromatin. Asterisk indicates that 5% FDR was used for this dataset. (B) Overlap analysis comparing the bound regions/genes identified in one dataset at high stringency with the bound regions/genes from the other dataset at lower stringency (25% FDR). Overlap is defined as at least 100 bp overlap between two bound regions. This analysis reveals considerable overlap in the data sets and we note, in particular, that 96% of the bound regions at 1% FDR in our data are also found in the Slattery et al. data at 25% FDR. (C) Correlation of windowed log2ratio scores along the whole genome for Ubx in haltere chromatin. (D) Correlation of windowed log2ratio scores along the whole genome for Hth in haltere chromatin. (0.75 MB TIF) [file pone.0014778.s001.tif]

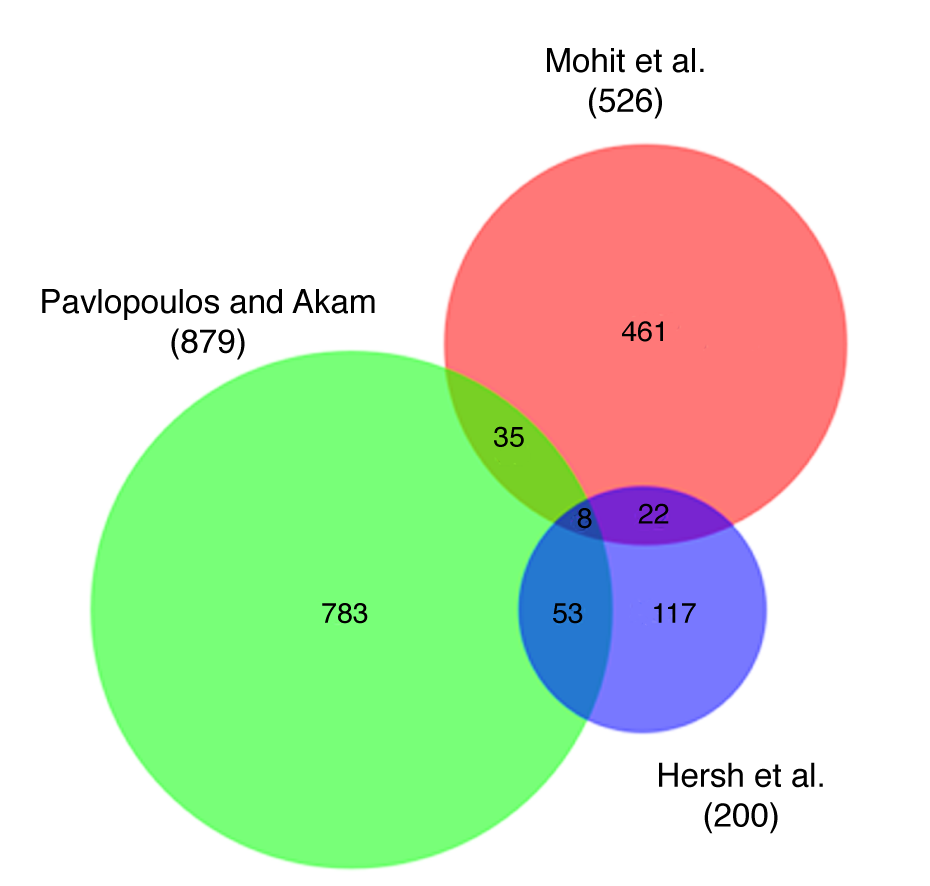

Supplement: Figure S2 — Overlap of differentially expressed genes identified in microarray experiments. Data from Hersh et al. [11], Mohit et al. [10] and combined timepoints from Pavlopoulos and Akam [42]. (0.23 MB TIF) [file pone.0014778.s002.tif]

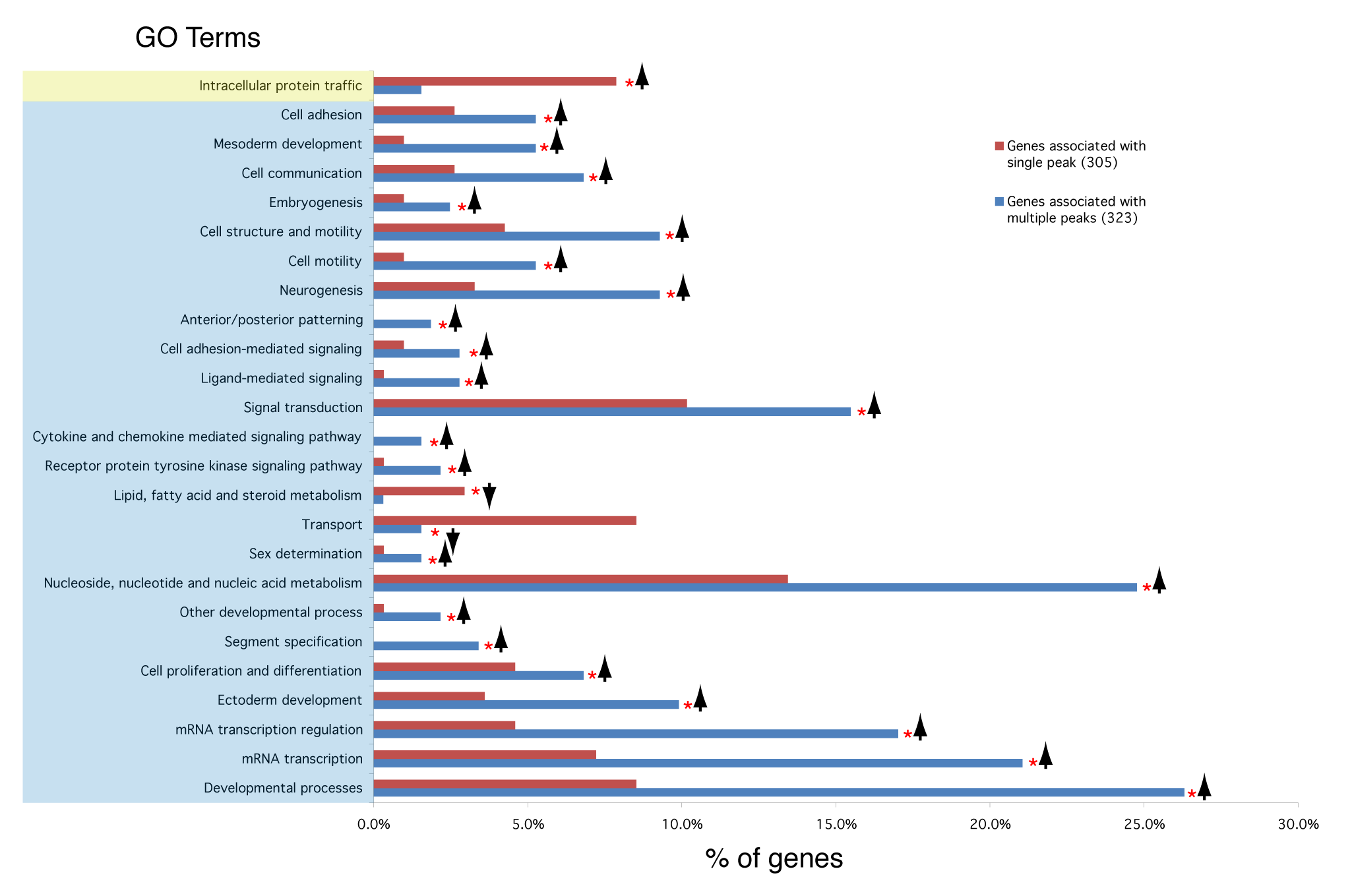

Supplement: Figure S3 — GO analysis of genes associated with multiple or single Ubx peaks. Red asterisks indicate significant over- or under-representation (p<0.05 Bonferroni corrected). Up arrows indicate over-representation, down arrows indicate under-representation. (0.46 MB TIF) [file pone.0014778.s003.tif]

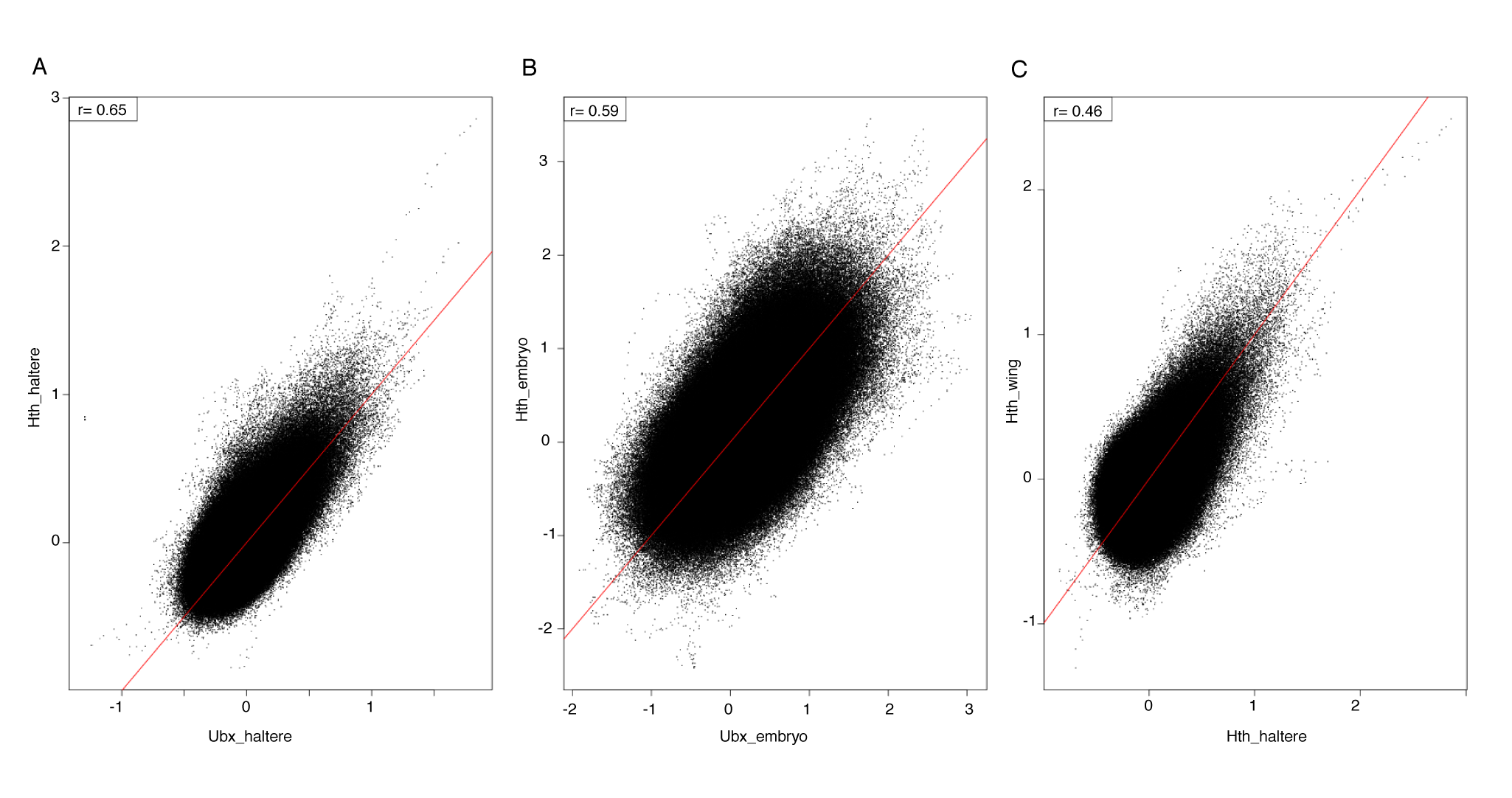

Supplement: Figure S4 — Hth versus Ubx binding: correlation analysis. Correlation of windowed log2ratio scores along the whole genome. (A) shows the correlation of the binding profiles of Hth versus Ubx in the haltere disc. In general, the genome-wide binding profiles of the two transcription factors are very similar (r = 0.65) in the haltere disc. (B) shows the correlation of the binding profiles of Hth versus Ubx in the embryo. (C) shows the correlation of the binding profiles of Hth in the wing disc versus Hth in the haltere disc. (0.59 MB TIF) [file pone.0014778.s004.tif]

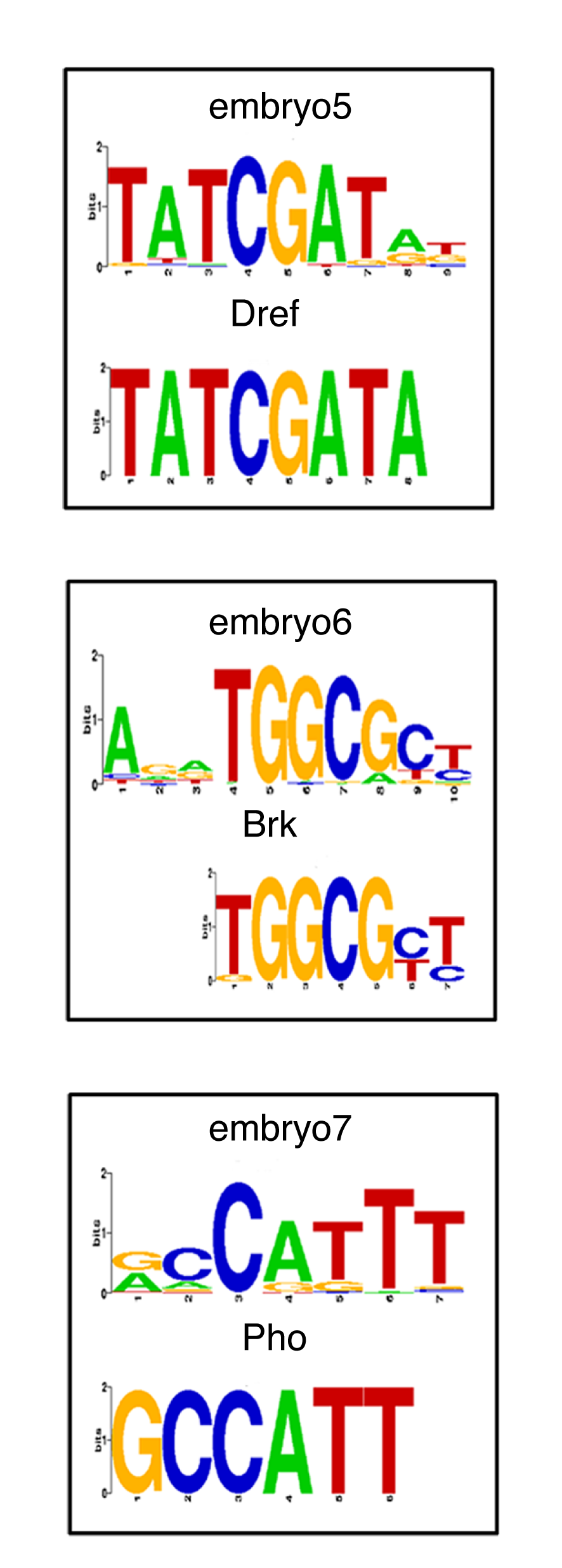

Supplement: Figure S5 — Candidate cofactor motifs. Enriched motifs derived from the Ubx and Hth ChIP-array data are compared to known motifs from the Drosophila Curated Transcription Factor Motifs database (http://www.bioinf.manchester.ac.uk/bergman/data/motifs/). (0.78 MB TIF) [file pone.0014778.s005.tif]
