# Supplementary material for: Genome-Wide Analysis of the Binding of the Hox Protein Ultrabithorax and the Hox Cofactor Homothorax in Drosophila
Source: PLoS One. 2011 Apr 5;6(4):e14778. doi: 10.1371/journal.pone.0014778 (PMC3071696; doi:10.1371/journal.pone.0014778)
Supplement: Table S1 — Number of bound regions across the genome and unique genes associated with bound regions for each of the proteins in the indicated chromatin source at a range of false discovery rates. For analysis of Ubx target genes the 1181 genes at 1% FDR in haltere disc chromatin were used however the histone gene repeats were removed giving a total of 1147 genes (see Table S2). Comparison of numbers of bound regions or gene sets across different chromatin sources is difficult due to signal/noise differences and consequent threshold effects. For a direct comparison of Hth and Ubx targets see Table 2. (0.03 MB DOC) [file pone.0014778.s006.doc]

| **Chromatin** | **Protein** | **Bound Regions** | | | **Unique Genes** | | |
| --- | --- | --- | --- | --- | --- | --- | --- |
|  |  | **1% FDR** | **10% FDR** | **25% FDR** | **1% FDR** | **10% FDR** | **25% FDR** |
| Embryo | Ubx | 124 | 554 | 1671 | 134 | 622 | 1754 |
|  | Hth | 34 | 2999 | 6514 | 31 | 2130 | 4049 |
| Haltere disc | Ubx | 1875 | 5699 | 9843 | 1181 | 3124 | 5103 |
|  | Hth | 6385 | 13901 | 21438 | 2804 | 5712 | 7910 |
| Wing disc | Hth | 1720 | 5868 | 10361 | 1001 | 3046 | 5032 |
